# Supplementary material for: Content-rich biological network constructed by mining PubMed abstracts
Source: BMC Bioinformatics. 2004 Oct 8;5:147. doi: 10.1186/1471-2105-5-147 (PMC528731; doi:10.1186/1471-2105-5-147)
Supplement: Additional File 2 — The original results of the above study (non-essential files are deleted to keep the file size under the limit set by BMC bioinformatics). [file 1471-2105-5-147-S2.bz2 › chilibotAdditionalFile2/dip30/306ID9117348E1304/html/CD44_CD9.html]

 


 **CD44** and **CD9** 
  
Found 109 abstracts in PubMed, retrieved 30.  
 

 What does Google say? 
 PDF only 
| .edu only 

---

**Interactive relationship** (e.g. stimulation, inhibition, etc)

**Neutral relationship**- Human multidrug resistance protein 1 MRP1  [ **CD9** ]  is a member of the ATP binding cassette ABC transport superfamily which also includes human multidrug resistance 1 MDR1 gene product P glycoprotein Pgp  [ **CD44** ] .  Ref: 12119019 Biochemistry, 2002

**Non-interactive relationship** (e.g. studied together, co-existance, homology, etc.)

- Nonetheless, Pgp  [ **CD44** ]  and MRP1  [ **CD9** ]  are detected in a significant proportion of untreated breast cancers on average 40 and 50% respectively, by immunohistochemistry, without a clear and consistent association with cancer stage.  Ref: 12653670 Endocr Relat Cancer, 2003
- P glycoprotein Pgp  [ **CD44** ]  and multidrug resistance protein MRP1  [ **CD9** ]  are the most important and widely studied members of the family that belongs to the ABC superfamily of transporters.  Ref: 12058813 Farmaco, 2002
- Different from Pgp  [ **CD44** ] , MRP1  [ **CD9** ]  contains an extra membrane spanning domain MSD1 with a putative extracellular amino terminus in addition to the core structure of two MSDs and two NBDs nucleotide binding domains.  Ref: 12119019 Biochemistry, 2002
- The multidrug resistance MR status of camptothecin CPT was investigated in colon adenocarcinoma HT29 cells, leukemia K562, and breast carcinoma MCF7 cells expressing P glycoprotein Pgp  [ **CD44** ]  and or MR associated protein MRP1  [ **CD9** ] .  Ref: 12115804 J Pharm Sci, 2002
- In this study we report on some basic characteristics of these sublines, namely, cross resistance to other anti cancer drugs investigated by in vitro MTT assay, expression of MDR associated proteins Pgp  [ **CD44** ] , MRP1  [ **CD9** ] , LRP, GST pi and Topo IIalpha as well as the functional activity of Pgp  [ **CD44** ]  and MRP.  Ref: 12584592 Neoplasma, 2002
- These include MDR1 P glycoprotein, PgP  [ **CD44** ] , the multidrug resistance related proteins MRP1  [ **CD9** ]  5, and lung resistance protein LRP.  Ref: 12699350 Curr Drug Targets, 2003
- Data obtained are presented in concurrence with Clusters of Thymic Epithelial Staining CTES classification for various monoclonal antibodies recognizing CD antigens CD1, CD1a, CD6,  **CD9** , CD14, CD16, CD29, CD30, CD32,  **CD44** , CD45RB, CD47, CD48, CD49a, CD49b, CD49c, CD49d, CD49e, CD49f, CD51, CD53, CD54, CD56, CD57, CD63, CD85, CD95, CD98, CD102, CD103, CD106, CD109, CD146, CD147, CD148, CD151, CD152, CD158a, CD158b, CD164, CD165, CD166 and for monoclonal antibodies 1B1 G7, A4, BD46, BLTZ, HP1C5, IND.64, M72, WU947 whose specifities are not yet defined.  Ref: 12725440 Ann Anat, 2003
- The compound is an extremely potent inhibitor of Pgp  [ **CD44** ]  mediated multidrug resistance MDR in cancer with an average FC50 of 32 nM, but does not inhibit multidrug resistance associated protein MRP1  [ **CD9** ] .  Ref: 11990779 Novartis Found Symp, 2002
- Overexpression of Pgp  [ **CD44** ] , MRP1  [ **CD9** ] , and LRP did not affect the in vitro activity of DX 8951f.  Ref: 12234607 Biochem Pharmacol, 2002
- A clonogenic assay was used to determine resistance, the expression of PGP  [ **CD44** ] , the multidrug resistance associated protein Mrp1  [ **CD9** ]  and topoisomerase IIalpha and beta were measured by Western blotting, and reverse transcriptase polymerase chain reaction was used for determination of mdr1a and b, and Mrp1  [ **CD9** ]  mRNA.  Ref: 12107549 Cancer Chemother Pharmacol, 2002
- Paraffin embedded samples of 13 pairs of primary untreated rhabdomyosarcomas and their residual, recurrent, or metastatic lesions after chemotherapy were assessed for expression of MDR proteins, including P glycoprotein Pgp  [ **CD44** ] , multidrug resistance associated protein MRP 1  [ **CD9** ] , and lung resistance related protein LRP.  Ref: 12612883 Hum Pathol, 2003
- RLIP76 does not meet the criteria for classical ABC proteins such as MRP1  [ **CD9** ]  or Pgp  [ **CD44** ] , but similar to ABC proteins, it has two ATP binding sequences, 69 GKKKGK 74 and 418 GGIKDLSK 425.  Ref: 12433796 Drug Metab Dispos, 2002
- Pgp  [ **CD44** ]  or MRP 1  [ **CD9** ]  expression did not change significantly, but LRP expression increased significantly after chemotherapy.  Ref: 12612883 Hum Pathol, 2003
- The analysis revealed the following altered expression such as up regulation of  **CD44** , keratins, and 14, aldehyde dehydrogenase,  **CD9**  and IP3 receptor type3.  Ref: 12402156 Br J Cancer, 2002
- PGP  [ **CD44** ] , LRP, and MRP1  [ **CD9** ]  expression at onset and at first relapse was low.  Ref: 12801292 Eur J Haematol, 2003
- Paraffin embedded sections from 56 previously untreated patients with metastatic TGCT were immunostained for Pgp  [ **CD44** ] , MRP1  [ **CD9** ] , BCRP, and LRP.  Ref: 12644825 Br J Cancer, 2003
- Efflux of the parent compound and metabolites out of cells by several drug transporters e.g., Pgp  [ **CD44** ] , BCRP, MRP1  [ **CD9** ] , MRP2 also occurs.  Ref: 12570720 Curr Med Chem, 2003
- The expression, of multidrug resistance MDR associated proteins PGP  [ **CD44** ] , LRP, MRP1  [ **CD9** ]  was also analyzed.  Ref: 12801292 Eur J Haematol, 2003
- Transport of xenobiotics and their metabolites by ATP binding cassette ABC transporters particularly P glycoprotein Pgp  [ **CD44** ]  and the multidrug resistance associated protein MRP1  [ **CD9** ]  has been extensively studied during last decade.  Ref: 12433796 Drug Metab Dispos, 2002
- The response rate was not affected by the overexpression of MDR related proteins PgP  [ **CD44** ] , MRP 1  [ **CD9** ] , and LRP.  Ref: 12224004 Ann Hematol, 2002
- Overexpression of MRP1  [ **CD9** ]  or Pgp  [ **CD44** ]  causes multidrug resistance in cancer cells.  Ref: 12119019 Biochemistry, 2002
- Overexpression of breast cancer resistance protein could be detected, both at the mRNA and protein level, while staining for Pgp  [ **CD44** ] , MRP1  [ **CD9** ] , or LRP was negative.  Ref: 12237778 Br J Cancer, 2002
- In MCF7 DoX cells, preferentially overexpressing Pgp  [ **CD44** ] , but also a significant level of MRP1  [ **CD9** ] , the IC 50 of CPT was 0.64 microM and thus presented a resistance index of 3.26 against CPT.  Ref: 12115804 J Pharm Sci, 2002
- To address whether multidrug resistance protein MRP affects GO susceptibility we characterized Pgp  [ **CD44** ] , MRP1  [ **CD9** ] , and MRP2 expression in CD33 cell lines and CD33 AML samples and analyzed the effect of the Pgp  [ **CD44** ]  inhibitor cyclosporine CSA and the MRP inhibitor MK 571 on GO induced cytotoxicity.  Ref: 12689934 Blood< MedlineTA>Blood, 2002
- These results further demonstrate that LY335979 is highly specific for Pgp  [ **CD44** ]  and does not modulate MRP1  [ **CD9** ]  or BCRP mediated resistance and can be used in combination with mitoxantrone and vinorelbine in tumor cells.  Ref: 12455064 Int J Cancer, 2003
- Intestinal transport of irinotecan in Caco 2 cells and MDCK II cells overexpressing efflux transporters Pgp  [ **CD44** ] , cMOAT, and MRP1  [ **CD9** ] .  Ref: 12065434 Drug Metab Dispos, 2002
- This study was undertaken to investigate the expression and predictive value for outcome of multidrug resistance associated MDR proteins P glycoprotein Pgp  [ **CD44** ] , MRP1  [ **CD9** ] , BCRP, and LRP, in advanced testicular germ cell tumours TGCT.  Ref: 12644825 Br J Cancer, 2003
- We found that 1 the NBD1s of Pgp  [ **CD44** ]  and MRP1  [ **CD9** ]  have ATPase and GTPase activities, 2 the K m s of Pgp  [ **CD44** ]  NBD1 for ATP and GTP hydrolysis are identical, while the K m of MRP1  [ **CD9** ]  NBD1 for ATP is lower than that for GTP, and 3 phosphorylation of MLD by PKA or PKC produces a marginal increase of V max for ATP hydrolysis, without affecting the affinity for ATP.  Ref: 12163030 Biochem Biophys Res Commun, 2002
- However, its cytotoxicity was altered by MRP1  [ **CD9** ] , but not Pgp  [ **CD44** ] , in naturally MRP1  [ **CD9** ]  expressing HT29 cells, and etoposide and doxorubicin resistant MCF7 VP and MCF7 DOX cells, respectively.  Ref: 12088114 Breast Cancer Res Treat, 2002
- Based on their immunophenotype, the cells are relatively homogeneous, with shared expression of integrin beta 1, the hyaluronate receptor  [ **CD44** ] , and the tetraspan protein  **CD9** , among other markers.  Ref: 12880371 Expert Opin Biol Ther, 2003
- The findings indicate that the in vivo expression of LRP, but not of Pgp  [ **CD44** ]  and MRP 1  [ **CD9** ] , is induced by chemotherapeutic treatment in rhabdomyosarcomas.  Ref: 12612883 Hum Pathol, 2003
- In this study, we compared the nucleotide triphosphatase activities of the isolated N terminal nucleotide binding domains NBD1 of Pgp  [ **CD44** ]  and MRP1  [ **CD9** ] , and explored the potential role of the phosphorylation target domain of Pgp  [ **CD44** ]  on the regulation of Pgp  [ **CD44** ]  NBD1 ATPase activity.  Ref: 12163030 Biochem Biophys Res Commun, 2002
- No correlation was found between Pgp  [ **CD44** ] , MRP1  [ **CD9** ] , and BCRP expression and response to chemotherapy and survival.  Ref: 12644825 Br J Cancer, 2003
- The present study was designed to investigate the intestinal absorption and efflux mechanisms of CPT 11 using in vitro cell culture models, Caco 2 cells, and engineered Madine Darby canine kidney MDCK II cells overexpressing P glycoprotein Pgp  [ **CD44** ] , canalicular multispecific organic anion transporter cMOAT, and multidrug resistance associated protein MRP1  [ **CD9** ] .  Ref: 12065434 Drug Metab Dispos, 2002
- Our study examines the ability of LY335979 Zosuquidar trihydrochloride to modulate 3 distinct ABC transporters that are mechanisms of drug resistance P glycoprotein Pgp  [ **CD44** ] , ABCB1, multidrug resistance associated protein MRP1  [ **CD9** ] , ABCC2 and breast cancer resistance protein BCRP, ABCG2.  Ref: 12455064 Int J Cancer, 2003
- The multidrug resistance proteins P glycoprotein Pgp  [ **CD44** ]  and MRP1  [ **CD9** ]  are drug efflux pumps.  Ref: 12163030 Biochem Biophys Res Commun, 2002
- BACKGROUND Efflux pumps situated on the plasma membrane, such as P glycoprotein Pgp  [ **CD44** ]  and the multidrug resistance related protein 1 MRP 1  [ **CD9** ] , have been shown to extrude HIV protease inhibitors from the cell.  Ref: 12218384 AIDS, 2002
- These results show efficient GTP hydrolysis by the NBD1s of Pgp  [ **CD44** ]  and MRP1  [ **CD9** ] , and a minor role of phosphorylation in the control of Pgp  [ **CD44** ]  NBD1 ATPase activity.  Ref: 12163030 Biochem Biophys Res Commun, 2002
- However, in the last decade considerable attention has been dedicated to the role played by membrane transporter proteins belonging to the ATP binding cassette protein superfamily, and in particular by the MDR1 product P glycoprotein Pgp  [ **CD44** ]  and the multidrug resistance protein MRP1  [ **CD9** ] .  Ref: 12653670 Endocr Relat Cancer, 2003
